# Hypoxia-induced up-regulation of miR-27a promotes paclitaxel resistance in ovarian cancer

**DOI:** 10.1042/BSR20192457

**Published:** 2020-03-31

**Authors:** Lanlan Feng, Fangrong Shen, Jinhua Zhou, Yan Li, Rong Jiang, Youguo Chen

**Affiliations:** 1 Department of Obstetrics and Gynecology, The Second Hospital of Taizhou, Taizhou City, Jiangsu Province 225500, China; 2 Department of Obstetrics and Gynecology, The First Affiliated Hospital of Soochow University, Suzhou, Jiangsu 215006, China; 3 Department of Obstetrics and Gynecology, The first People’s Hospital of Yancheng City, Jiangsu Province 224000, China

**Keywords:** APAF1, HIF-1α, hypoxia, miR-27a, ovarian cancer, paclitaxel resistance

## Abstract

Ovarian cancer (OC) is a malignant tumor with high mortality in women. Although cancer patients initially respond to paclitaxel chemotherapy following surgery, most patients will relapse after 12–24 months and gradually die from chemotherapy resistance. In OC, cancer cells become resistant to paclitaxel chemotherapy under hypoxic environment. The miR-27a has been identified as an oncogenic molecular in ovarian cancer, prostate cancer, liver cancer etc. In addition, the miR-27a is involved in hypoxia-induced chemoresistance in various cancers. However, the role of miR-27a in hypoxia-induced OC resistance remains unclear. The aim of the present study was to investigate the regulatory mechanism of miR-27a in hypoxia-induced OC resistance. The expression of HIF-1α induced Hypoxia overtly up-regulated. At the same time, hypoxia increased viability of Skov3 cells and decreased cell apoptosis when treated with paclitaxel. The expression of the miR-27a was obviously up-regulated under hypoxia and involved in hypoxia-induced paclitaxel resistance. Follow-up experiments portray that miR-27a improved paclitaxel resistance by restraining the expression of APAF1 in OC. Finally, we further elucidated the important regulatory role of the miR-27a-APAF1 axis in OC through *in vivo* experiments. According to our knowledge, we first reported the regulation of miR-27a in hypoxia-induced chemoresistance in OC, providing a possible target for chemoresistance treatment of OC.

## Introduction

Ovarian cancer is the most frequent malignant genital tumor and one of the lethal factors of gynecological malignancy [1]. Chemotherapy is one of the main treatments for OC, with predominant initial response rate. However, early chemotherapy is effective in the treatment of OC, but it is easy to relapse and eventually develop drug resistance [2]. Paclitaxel is a primary chemotherapy against many cancers including OC, the chemotherapy resistance is a major limiting factor in its clinical success [3]. Therefore, there is an urgent need to find therapeutic targets related to chemotherapy resistance and disease recurrence.

It is well known that hypoxia induces resistance among various tumors. Previous reports showed that hypoxia can enhance drug resistance in non-small lung cancer cells [4]. Hypoxia-inducible factors are involved in chemotherapy resistance of breast cancer stem cells [5]. The study of hypoxia in paclitaxel resistance of OC has also been reported. For example, previous study has found that hypoxia microenvironment promotes chemotherapeutic resistance of OC by influencing cell cycle through c-Src [6]. Nevertheless, the regulatory mechanism of hypoxia-induced drug resistance is not very clear, which has gradually become the spotlight of research.

MiRNAs are a group of endogenous non-coding RNAs that consist of 19–25 nucleotides with diverse functions. The miRNAs regulate gene expression by inhibiting translation and other means [7]. MiR-27a is involved in the drug resistance of many tumors. MiR-27a is involved in the process of cisplatin resistance and metastasis in lung cancer [8]. MiR-27a also involved in leukaemia chemoresistance [9]. The role of miR-27a in OC has also been reported. For instance, miR-27a promotes epithelial–mesenchymal transition (EMT) in OC by regulating FOXO1 expression [10]. MiR-27a also regulates the chemosensitivity of human OC cell lines by targeting Cullin 5 [11]. Previous report has also found that miR-27a was involved in gastric cancer hypoxia-induced chemoresistance [12]. Nevertheless, studies on miR-27a in hypoxia-induced chemoresistance of OC have not been reported.

In the present study, miR-27a was found to play an critical role in tumor chemoresistance by detecting the expression of miR-27a in hypoxia and normoxia. The hypoxia environment up-regulates the expression of miR-27a via the HIF-1α pathway, and up-regulation of miR-27a promotes tumor chemoresistance. Subsequently, we used TargetScan software to predict a target for miR-27a-APAF1, which is an apoptosis-related protein. Then we found that the protein is indeed down-regulated under hypoxia. We also demonstrated that APAF1 is a target of miR-27a by luciferase reporter assay and Western blot. Finally, our study further found that miR-27a regulates hypoxia resistance in ovarian cancer through APAF1.

## Materials and methods

### Cell lines and culture conditions

Skov3, A2780, HO8910, OVCAR3 and PEO-1 (human ovarian cancer) cells were purchased from the Chinese Academy of Sciences, Shanghai Institute of Biochemistry and Cell Biology (Shanghai, China). These cells were grown in RPMI-1640 (10% FBS).

Mice were obtained from Charles River (Beijing, China). All mice were kept on a 12-h light–dark cycle in room temperature with a free access to food and water. Mice were anesthetized with an intraperitoneal injection of 50 mg/kg pentobarbital sodium (Synthgene, Nanjing, China).Sacrifice was performed by intraperitoneal injection of sodium pentobarbital (50 mg/kg) followed by cervical dislocation, and mortality was confirmed when no spontaneous breathing for 2–3 min and no blinking reflex were observed.

### Cell treatments

Hypoxia treatment: For all experiments involving hypoxia (and the respective normoxia controls), cells were seeded at a given density and were always permitted to attach for 16–20 h in atmospheric oxygen before exposure to hypoxia. Cells were maintained at 37°C in humidified incubator containing 20% O_2_, 5% CO_2_ and 75% N_2_ in normoxia. Hypoxia condition were achieved at 37°C by culturing cells in a modified incubator chamber flushed with a gas mixture containing 1% O_2_, 94% N_2_ and 5% CO_2_ in a humidified atmosphere.

Paclitaxel treatment: Paclitaxel was purchased from Sigma-Aldrich. The drugs were kept as 1 mg/ml stock solutions in sterile PBS at −20°C. For cell viability measurement, tumor cells were treated for 7 days at a concentration of 0, 4, 8 and 12 μM. The culture medium was replaced on a daily basis. For *in vivo* therapy, the nude mice were injected once every 3 days with 5mg/kg of paclitaxel (dissolved in normal saline)/kg body weight. Controls were treated with the same volume of normal saline.

### RNA extraction and qRT-PCR

The extraction of total RNA and the analysis of qRT-PCR were performed according to the previous description [13]. We used TRIZOL reagent (Thermofisher, U.S.A.) to extract total RNA by in cells and tissues. Taqman probes (Applied Biosystems, U.S.A.) were used to quantify miRNAs. Briefly, 1 µg of total RNA was transcribed to cDNA using AMV reverse transcriptase (Takara, Japan) and a RT primer. The reaction conditions were: 16°C for 30 min, 42°C for 30 min and 85°C for 5 min. Real-time PCR was performed using a Taqman PCR kit on an Applied Biosystems 7300 sequence detection system (Applied Biosystems, U.S.A.). The reactions were performed in a 96-well plate at 95°C for 10 min, followed by 40 cycles of 95°C for 10 s and 60°C for 1 min. U6 was used as the internal control. The qRT-PCR primers sequences are: miR-27a-F: 5′-GCGCGTTCACAGTGGCTAAG-3′ miR-27a-R: 5′- AGTGCAGGGTCCGAGGTATT -3′. All primers are designed by primer5 software.

### Western blotting analysis

The Skov3 cells were washed twice with PBS (ice-cold) and centrifuged at 12000 ***g*** for 10 min at 4°C; lysis was performed using RIPA lysis buffer (Synthgene, China) and incubated on ice for about 30 min. Cell lysates were centrifuged for another 10 min at 4°C (12000 ***g***). Subsequently, determination of protein concentration in supernatant by BCA protein Kit (Synthgene, China). The protein was then incubated overnight with the following primary antibodies at 4 C: HIF-1α, APAF1, β-actin. β-Actin served as a loading control and protein bands were quantified using ImageJ Software.

### Cell viability assay

MTT assay was used to detect the sensitivity of cells to paclitaxel. The implementation method refers to previous study [14].

### Cell apoptosis assay

Flow cytometry was used to detect cell apoptosis with FITC and PI detection kits (BestBio, Shanghai China) and performed according to standard operating methods. The implementation method refers to previous report [13].

### Plasmid construction and luciferase reporter assay

In short, the 3′-UTR sequence of APAF1 is searched from the NCBI. And the 3′-UTR of APAF1 that contained the presumed miR-27a binding sites (https://www.targetscan.org). We used pMIR-REPORT Luciferase vector (Ambion) to construct pMIR-APAF1-3′-UTR plasmid. The implementation method refers to previous study [13].

### miRNA transfection

The mimics and inhibitors of miR-27a were chemically synthesized by GenePharma Co., Ltd. (Shanghai, China). Skov3 and Skov3/ paclitaxel cells were seeded in 6-well plates at 3 × 10^5^ cells/well and cultured for 18 h. The cells were then transfected with 100 pmol of the miR-27a mimics, inhibitors or negative control (NC) RNA using Lipofectamine 2000 and Opti-MEM I reduced serum medium (Invitrogen, Carlsbad, CA, U.S.A.), according to the manufacturer’s instructions.

### siRNA preparation and transfection

HIF-1α knockdown was accomplished by transfecting cells with siRNA. HIF-1α and control siRNA were synthesized by Synthgene (China). The implementation method refers to previous report [15]. SiRNA transfections were performed according to the manufacturer’s instructions using Lipofectamine 2000 reagent. After that, the transfected system was removed, and it was not until 24 h cultured in normal media were the cells used for further experiment.

### APAF1 overexpression plasmid

Control plasmid (pCMV6) and overexpression plasmid (pCMV6-APAF1) came from Synthgene (China). The full-length sequence of APAF1 can be found in the Supplementary Data.

### Establishment of tumorigenicity assay in mice

Animal care and killing were carried out with the approval of the Institutional Animal Care and Use Committee (IACUC) of Soochow University. Skov3 cells were transfected with a miR-27a mimic, or co-transfected with a miR-146a mimic and a APAF1 OE plasmid. The Skov3 cells were then injected subcutaneously into the right back flank of 4- to 5-week-old female nude mice (Nanjing Biomedical Research Institute of Nanjing University) (5 × 10^6^ cells/mouse, 5 mice/group). We measured the growth curve of the tumors, the size of the tumors and the expression of AFAP1 protein.

### Statistical analyses

The results were expressed as the mean ± standard deviation of the mean of three independent experiments. Comparisons were determined using Student’s *t*-test and a *P* < 0.05 was considered statistically significant.

## Results

### Hypoxia increases paclitaxel resistance in OC

**Figure 1:**
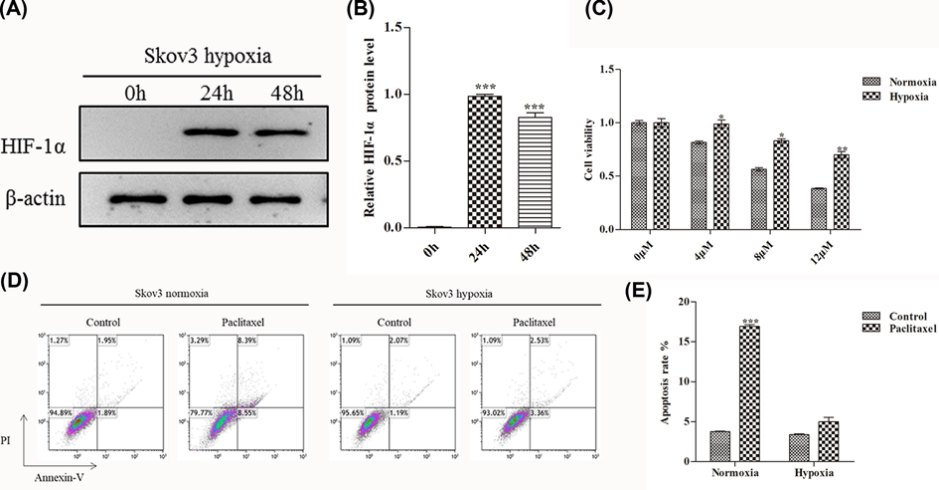
Hypoxia increases cell viability and reduces apoptosis in OC (**A**) WB analysis the expression of HIF-1α protein in Skov3 cells when hypoxia treatment for 0, 24 and 48 h. (**B**) Quantify the protein bands of the HIF-1α protein (O.D. ratio over β-actin). (**C**) Cell viability of Skov3 cells was measured by MTT assay. Skov3 cells were treated with 0, 4, 8 and 12 μM paclitaxel under the condition of hypoxia or normoxia. (**D** and **E**) Flow cytometric analysis of skov3 cell apoptosis rate when treated with paclitaxel (4 μM, 48 h) under the condition of hypoxia or normoxia. Data are shown as mean ± SEM (*n* = 3). Asterisks indicate significant differences from the control (*, *P* <0.05; **, *P* <0.01; ***, *P* <0.001, Student’s *t*-test).

Paclitaxel is one of the most effective chemotherapy drugs for numerous malignancies, including cervical cancer, ovarian cancer, breast cancer and so on. However, tumors are susceptible to paclitaxel resistance after chemotherapy and hypoxia is one of the causes of paclitaxel resistance in tumors [6,14,15]. Recent report showed that hypoxia inducible factor-1α (HIF-1α) was over-expressed in most OC patients under hypoxia stress, which is a key regulator of hypoxia [16]. Hence, we first treated Skov3 cells (OC cells) with hypoxia and used WB assay to detect HIF-1α protein expression after 0, 24 and 48 h treatment. Over time, HIF-1α expression was significantly up-regulated and peaked at 24 h and slightly down-regulated at 48 h (Figure 1A,B). Subsequently, we examined cell viability of Skov3 cells when treated with paclitaxel by MTT assay. Compared with normoxia group, the cell viability of hypoxia group was significantly up-regulated when treated with different concentrations of paclitaxel (0, 4, 8 and 12 μM) (Figure 1C). The apoptotic rate was further detected by flow cytometry. Compared with paclitaxel treatment, the percentage of apoptotic cells in normoxia group was strikingly higher than the control (without paclitaxel treatment). However, paclitaxel treatment had little effect on apoptosis in hypoxia group when compared with the control (without paclitaxel treatment) (Figure 1D,E).

### Hypoxia increases miR-27a expression through HIF-1α and hypoxia promotes paclitaxel resistance through regulating miR-27a

**Figure 2:**
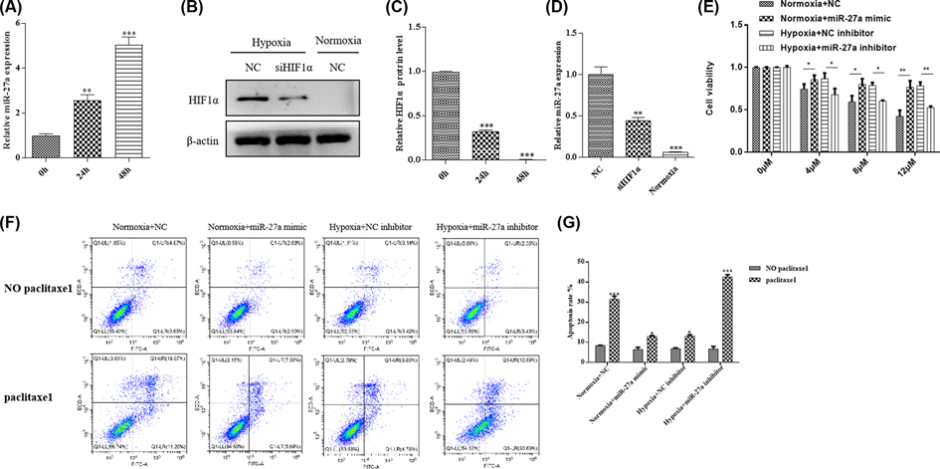
Hypoxia increases the expression of miR-27a by HIF-1α and promotes paclitaxel resistance (**A**) The expression level of *miR-27a* was detected by qPCR in Skov3 cells when hypoxia treatment for 0, 24 and 48 h. The *miR-27a* expression level in Skov3 cells when hypoxia treatment for 0 h was set as reference (was set to 1). (**B**) HIF-1α protein expression in NC or siHIF-1α Skov3 cells under the condition of hypoxia or normoxia. (**C**) Quantify the protein bands of the HIF-1α protein (O.D. ratio over β-actin). (**D**) The expression of *miR-27a* in in NC or siHIF-1α Skov3 cells under the condition of hypoxia or normoxia. (**E**) Cell viability was determined by MTT assay. The Skov3 cells were added 0, 4, 8, 12 μM paclitaxel under the condition of hypoxia (transfected with or without miR-27a inhibitors) or normoxia (transfected with or without miR-27a mimics). (**F** and **G**) Flow cytometric analysis of skov3 cell apoptosis rate when treated with paclitaxel (4 μM, 48 h) under the condition of hypoxia (transfected with or without miR-27a inhibitors) or normoxia (transfected with or without miR-27a mimics). Data are shown as mean ± SEM (*n* = 3). Asterisks indicate significant differences from the control (*, *P* <0.05; **, *P* <0.01; ***, *P* <0.001, Student’s *t*-test).

Previous study has reported that high expression of miR-27a is linked with chemosensitivity, cancer proliferation and invasion in OC [11]. The miR-27a has also been found to be involved in hypoxia induction of chemotherapy resistance in gastric cancer [12]. Therefore, we measured the *miR-27a* expression first in Skov3 cells when hypoxia treatment. The expression of *miR-27a* increased by 1.6- and 4.1-fold, respectively when hypoxia treatment for 24 and 48 h, compared with the untreated cells (treated 0h) (Figure 2A). We also detected miR-27a expression in other OC cell lines (including A2780, HO8910, OVCAR3 and PEO-1) during hypoxia treatment. The results were consistent with the changes in Skov3 cells (Supplementary Figure S1). Of these five cell lines, miR-27a was most significantly up-regulated in SKOV3 cells, so this cell line was selected for subsequent *in vitro* experiments. These results indicate that miR-27a is involved in the regulation of OC under hypoxia.

We next sought to confirm this hypothesis. So, we knocked down *HIF-1α* via siRNA interference technology. Then, we examined HIF-1α expression by using WB. In hypoxia, HIF-1α expression level was down-regulated in the siHIF-1α cell lines, whereas HIF-1α was not expressed in normoxia (Figure 2B,C). Simultaneously, we detected the *miR-27a* expression level by qPCR, and the change trend was consistent with the result of HIF-1α protein (Figure 2D). These findings delineate that hypoxia increases miR-27a expression through the pathway of HIF-1α.

In order to ulteriorly demonstrate the role of miR-27a in paclitaxel resistance of Skov3 cell. We first transfected miR-27a inhibitor and mimic (knockdown and over-expression of miR-27a, respectively) into Skvo3 cells. Then we detected the cell viability of Skvo3 cells when added different amounts of paclitaxel. As you can see in Figure 2E, with the increase of paclitaxel concentration (0, 4, 8 and 12 μM), the cell viability of Skvo3 cells in the normoxia group (transfected with NC) were gradually decreased. However, when the normoxia group cells were transfected with miR-27a mimic, the cell viability of cells were significantly increased. Compared with the normoxia group cells, the result of Figure 1C has been shown that the cell viability was significantly raised in the hypoxia group when treated with different concentrations of paclitaxel. However, when the hypoxia group cells were transfected with miR-27a inhibitor, the cell viability of cells was significantly decreased when compared with the hypoxia group (transfected with NC inhibitor) (Figure 2E).

Finally, we also measured the apoptotic rate of the Skvo3 cells. In the normoxia group (transfected with NC), paclitaxel treatment significantly increased the apoptotic rate, while the apoptotic rate decreased significantly when transfected with miR-27a mimic. However, compared with the hypoxia group (transfected with NC inhibitor), the apoptotic rate of hypoxia group cells increased significantly when transfected with miR-27a inhibitors (Figure 2F,G). All these results elucidate that hypoxia promotes paclitaxel resistance through regulating miR-27a.

### APAF1 is a direct target of miR-27a in OC

**Figure 3:**
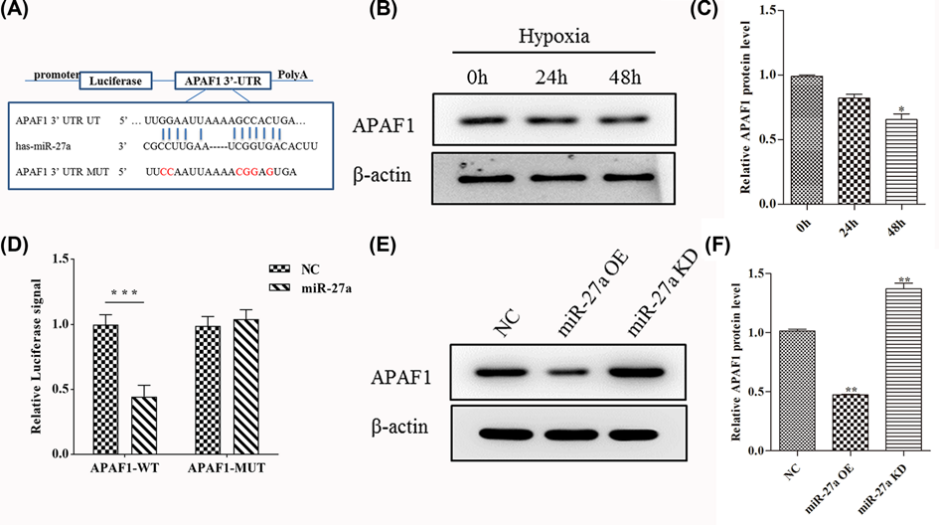
MiR-27a regulates gene expression of its target *APAF1* (**A**) 3′-UTR base pairing diagram of miR-27a and APAF1. Replacement of Guanine base with Cytosine (G to C) or replacement of Cytosine bases with Guanine (C to G) can also be used for the construction of mutant reporter. (**B**) The expression of APAF1 protein in Skov3 cells by Western blot when hypoxia treatment for 0, 24 and 48 h. (**C**) Quantify the protein bands of the HIF-1α protein (O.D. ratio over β-actin). (**D**) Cells were co-transfected with miR-27a mimics and a luciferase reporter containing a fragment of the APAF1 3′-UTR harboring either the miR-27a binding site (APAF1-3′-UTR-WT) or a mutant (APAF1-3′-UTR-MUT). (**E** and** F**) The APAF1 protein expression in the miR-27a OE and miR-27a KD cells by Western blot analysis. Data are shown as mean ± SEM (*n* = 3). Asterisks indicate significant differences from the control (*P <0.05; **P <0.01; ***P <0.001, Student’s *t*-test).

We redouble explored how miR-27a participates in hypoxia-induced paclitaxel resistance of OC. The major component of apoptosome contains apoptotic protease activating factor 1 (APAF1) [17]. A myriad of recent research have found that APAF1 plays an important role in ovarian cancer cell proliferation and chemotherapy resistance [13,18]. Recent reports have also shown that miR-27a suppresses APAF1 and alleviate hypoxia-induced neuronal apoptosis [19]. Consequently, we used the TargetScan database to predict possible targets for miR-27a in OC. *APAF1* gene was found to contains one putative site of the 3′-UTR untranslated region (3′-UTR) that matched to the miR-27a seed region (Figure 3A). Then, we examined the expression of APAF1 when 0, 24 and 48 h hypoxia treatment. Compared with 0 h treatment, the expression of APAF1 significantly decreased at 48 h treatment (Figure 3B,C). We also measured the protein content of APAF1 in A2780, HO8910, OVCAR3 and PEO-1 cell lines when 0, 24 and 48 h hypoxia treatment, and the results were consistent with the change trend in Skov3 cells (Supplementary Figure S2). This result suggest that APAF1 responds to hypoxia in OC.

We further set up the luciferase reporter plasmid (containing the wild-type (WT) and mutation-type 3′-UTR) of target gene APAF1 using luciferase reporter vector. We first used the transfection reagent to transfect these reporter gene plasmids (WT and MUT) into Skov3 cells together with the mimics of miR-27a. Compared with negative control (NC), the addition of miR-27a mimics significantly reduced WT reporter activity. In sharp contrast with WT reporter activity, the addition of miR-27a mimics did not affect the activity of mutant reporter activity (Figure 3D). Finally, we measured the expression of APAF1 when Skov3 cells were transfected miR-27a inhibitors and mimics. Compared with NC cells, the expression of APAF1 significantly decreased in miR-27a OE cells, but the expression of APAF1 significantly increased in miR-27a KD cells (Figure 3D,E). These results demonstrate that miR-27a targets APAF1 and is involved in hypoxia regulation of OC.

### MiR-27a induced down-regulation of APAF1 contributes to paclitaxel resistance in OC cells *in vitro*

**Figure 4:**
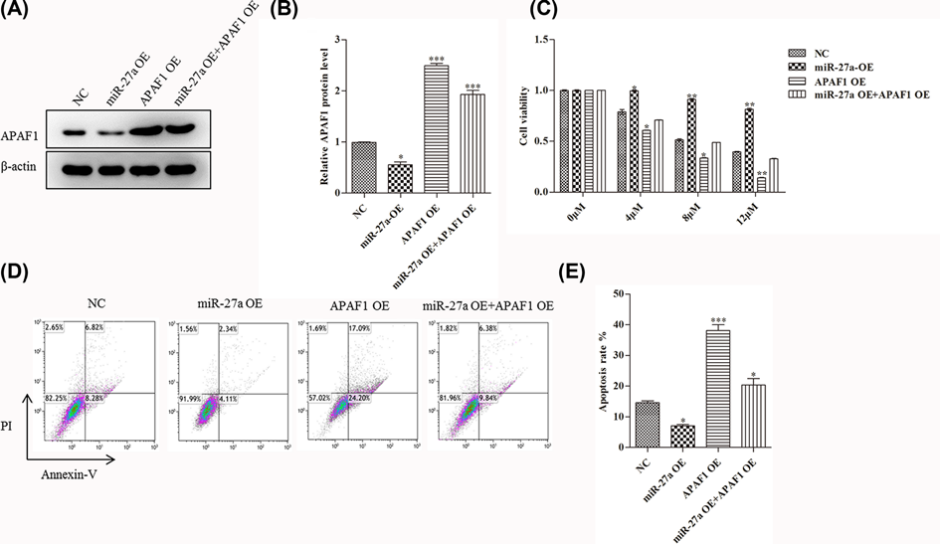
MiR-27a improves OC paclitaxel resistance via APAF1 *in vitro* (**A** and **B**) The APAF1 protein expression in the miR-27a OE, APAF1 OE, both miR-27a and APAF1 OE Skov3 cells by Western blot analysis, respectively. (**C**) Cell viability was measured by MTT assay. Skov3 cells were treated with 0, 4, 8, 12 μM paclitaxel in miR-27a OE, APAF1 OE, both miR-27a and APAF1 OE Skov3 cells, respectively. (**D** and** E**) Flow cytometric analysis of skov3 cell apoptosis rate in miR-27a OE, APAF1 OE, both miR-27a and APAF1 OE Skov3 cells, respectively. Data are shown as mean ± SEM (*n* = 3). Asterisks indicate significant differences from the control (*, *P* <0.05; **, *P* <0.01; ***, *P* <0.001, Student’s *t*-test).

In order to further explore the role of APAF1 in the regulation of paclitaxel resistance in OC under hypoxia by miR-27a. First, we tested the APAF1 expression level in miR-27a OE, APAF1 OE and both miR-27a and APAF1 OE Skvo3 cells. As shown below in Figure 4A, compared with NC, the expression of APAF1 strikingly decreased in miR-27a OE cells. When over-expressing APAF1 or over-expressing both miR-27a and APAF1, the expression level of APAF1 were all predominantly up-regulated (Figure 4A,B).

Then, we detected the cell viability of Skvo3 cells when treated with different concentrations of paclitaxel. As shown below in Figure 4C, with the increase of paclitaxel concentration, the cell viability of the miR-27a OE Skvo3 cells were overtly higher than that of NC cells. While the cell viability was significantly decreased in APAF1 OE cells. Besides, the cell viability were also strikingly decreased in both miR-27a and APAF1 OE cells.

Finally, we also examined the apoptotic rate. As shown in Figure 4D,E, compared with NC cells, the cell apoptotic rates of the miR-27a OE Skvo3 cells were predominantly decreased. The cell apoptotic rates were significantly increased in APAF1 OE cells. However, the cell apoptotic rates were strikingly decreased in both miR-27a and APAF1 OE cells. All these results illustrate that miR-27a is involved in hypoxia-induced paclitaxel resistance of OC through APAF1.

### MiR-27a induced down-regulation of APAF1 contributes to paclitaxel resistance in OC *in vivo*

**Figure 5:**
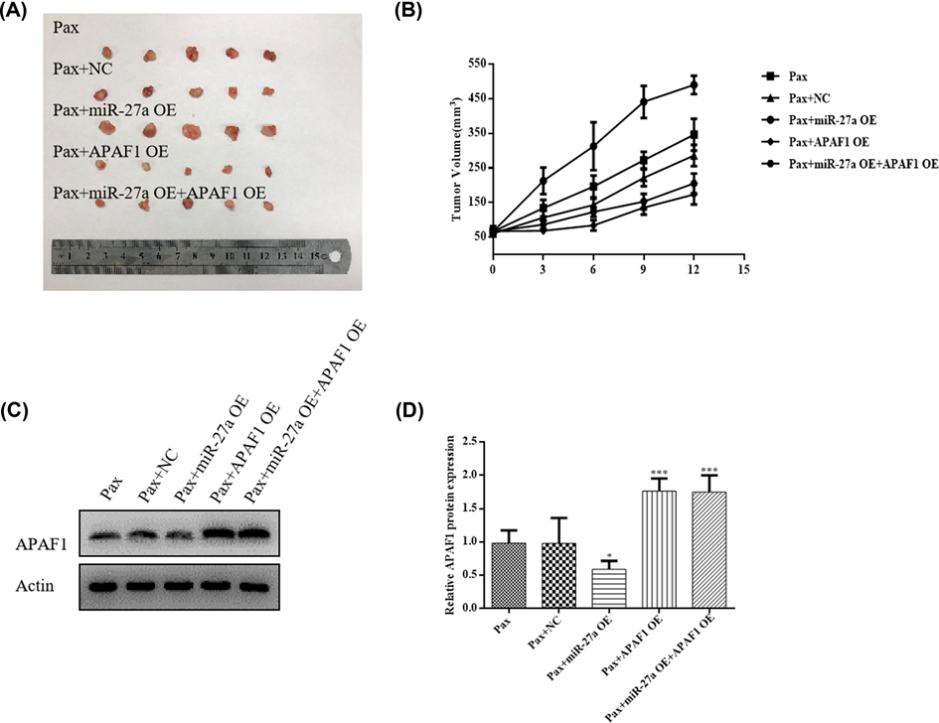
MiR-27a improves OC paclitaxel resistance via APAF1 *in vivo* (**A**) Optical images of tumor tissues dissected from Huh-7 tumor-bearing mice after 16 days. (**B**) Relative tumor growth curves. (**C** and **D**) Western blotting analysis the expression of APAF1 protein in tumor tissue from Skov3 tumor-bearing mice. Data are shown as mean ± SEM (*n* = 3). Asterisks indicate significant differences from the control (*P <0.05; **P <0.01; ***P <0.001, Student’s *t*-test).

In Figure 4A–F, we found that miR-27a regulates chemoresistance of OC via AFAP1 *in vitro*. Therefore, we further evaluated that whether miR-27a could regulates chemoresistance of OC *in vivo*. Skov3 cells (all cells treated with paclitaxel) were transfected with miR-27a mimic, APAF1 OE plasmid and co-transfected with miR-27a mimic and APAF1 OE plasmid and implanted subcutaneously into severe combined immunodeficiency mice to allow tumor formation. As shown in Figure 5A, the xenograft tumors were significantly bigger in the miR-27a-OE mice group when compared with the PAX group or PAX group (transfected with NC). In sharp contrast, the tumors were significantly smaller in the APAF1-OE mice group when compared with the PAX group or PAX group (transfected with NC). However, simultaneous APAF1-OE alleviated the effect of miR-27a on tumor growth. In addition, tumor growth curves confirms this result (Figure 5B). These results suggest that miR-27a may promotes tumor growth of OC by inhibiting APAF1.

Finally, we also examined APAF1 protein expression by WB in tumor tissue from Skov3 tumor-bearing mice. As shown in Figure 5C,D, the expression of APAF1 were significantly down-regulated in the miR-27a-OE mice group when compared with the PAX group or PAX group (transfected with NC). However, the expression of APAF1 were significantly up-regulated in the APAF1-OE mice group or miR-27a OE+ APAF1 OE group when compared with the PAX group or PAX group (transfected with NC). These results are consistent with *in vitro* experiments. In conclusion, both *in vitro* and *in vivo* experiments have further validated that miR-27a may promotes paclitaxel resistance of OC by inhibiting APAF1.

## Discussion

OC is one of the most lethal disease among all gynecological malignancies [20]. Resistance of OC to existing drugs is a huge challenge that urgently needs to be addressed, which further exacerbates recurrence and mortality [21]. As a result, exploring the drug resistance mechanism of OC and studying new targets for cancer regulation have gradually become the focus of scientific research. In this article, we confirmed the mechanism of hypoxia-induced OC paclitaxel resistance.

Previous study has found that TMEM45A plays a crucial role in hypoxia-induced breast cancer and liver cancer chemotherapy resistance [22]. Anterior research has also reported that the miR-21 could changes paclitaxel sensitivity and affects the expression of HIF-1α in OC [20]. All these results suggest that hypoxia can induce cancer tolerance to chemotherapeutic drugs. However, the mechanism of hypoxia-induced drug resistance remains unclear. Therefore, we first examined the resistance of OC to paclitaxel in hypoxia. HIF-1α expression was significantly up-regulated during hypoxia induction in Skov3 cells (Figure 1A,B). Compared with the normoxia group cells, the cell viability of Skov3 cells in hypoxia group was significantly increased when different concentrations of paclitaxel were added (Figure 1C). We also used flow cytometry to detect apoptosis. In the normoxia group, the apoptosis rate was predominantly increased after the addition of paclitaxel compared with the untreated control. However, in the hypoxia group, paclitaxel treatment had little effect on the apoptotic rate of cells (Figure 1D,E). These data are consistent with previous explorations.

Previous reports have shown that miR-27a plays an important role in hypoxia-induced drug resistance in many tumors. For instance, miR-27a regulates hypoxia-induced chemotherapy resistance in lung cancer, leukaemia and gastric cancer [8,9,12]. However, the function of miR-27a in hypoxia-induced drug resistance in ovarian cancer has not been reported. Accordingly, we tested the expression of *miR-27a* in paclitaxel treatment. The expression of *miR-27a* was significantly up-regulated after paclitaxel treatment (Figure 2A). To further demonstrate the function of miR-27a in OC under hypoxia, we knocked down *HIF-1α*. First, we detected the expression of HIF-1α after knocking down *HIF-1α*. In hypoxia group, compared with NC Skov3 cells, the expression of HIF-1α protein was overtly down-regulated in siHIF-1α Skov3 cells (Figure 2B,C). Consistent with this, the expression of *miR-27a* was also significantly down-regulated when *HIF-1α* knockdown (Figure 2D). Then, we transfected miR-27a inhibitors and mimics into Skov3 cells and tested cell viability. Compared with untreated cells, the cell viability was significantly reduced in the hypoxic group (transfected with miR-27a inhibitors) and significantly increased in the normoxic group (transfected with miR-27a mimics) under different concentrations paclitaxel treatment. Finally, the apoptotic rate was measured, and the trend of the results was consistent with the cell viability (Figure 2F,G).

We next sought to explore the mechanism of miR-27a in the regulation of hypoxia-induced drug resistance. We use the appropriate software to find possible targets for miR-27a (TargetScan 5.1 database). Through software screening, APAF1 protein was found to bind to miR-27a (Figure 3A). Previous reports have shown that APAF1 is a key regulator of apoptosis and plays an important role in chemotherapeutic resistance of melanoma [23]. Exosome-mediated miR-21 regulates paclitaxel resistance through APAF1 in ovarian cancer [18]. Therefore, we detected the expression of APAF1 protein during hypoxia induction. The results showed that the expression of APAF1 was significantly down-regulated after 48 h of hypoxia induction when compared with 0 h treatment (Figure 3B). We further set up the luciferase reporter plasmid of target gene *APAF1* using luciferase reporter vector. Compared with negative control (NC), the addition of miR-27a mimics significantly reduced WT reporter activity. In sharp contrast with WT reporter activity, the addition of miR-27a mimics did not affect the activity of mutant reporter activity. (Figure 3D). In the meantime, we measured the expression of APAF1 when Skov3 cells were transfected miR-27a inhibitors and mimics. The results indicate that miR-27a negatively regulates the expression of APAF1 protein (Figure 3E,F).

To further elucidate the role of APAF1 in the regulation of hypoxia-induced drug resistance by miR-27a. We then added APAF1 function replenishment experiments. First, we measured the APAF1 expression in miR-27a OE, APAF1 OE and both miR-27a and APAF1 OE Skvo3 cells. Then, we detected cell viability and apoptosis of these cells. Finally, we further verified it by *in vivo* experiments. All these data indicate that miR-27a regulates the APAF1 expression when added paclitaxel treatment.

All in all, within our cognitive range, in this paper, we first explored the mechanism of miR-27a regulating OC paclitaxel resistance induced by hypoxia through APAF1. This will provide useful reference and help for the treatment of drug resistance in OC chemotherapy.

## Supplementary Material

Supplementary Figures S1-S2

## Abbreviations

APAF1: apoptotic protease activating factor 1
EMT: epithelial–mesenchymal transition
HIF-1α: hypoxia inducible factor-1α
OC: ovarian cancer
