# Supplementary material for: Hypoxia-induced up-regulation of miR-27a promotes paclitaxel resistance in ovarian cancer
Source: Biosci Rep. 2020 Mar 31;40(4):BSR20192457. doi: 10.1042/BSR20192457 (PMC7109003; doi:10.1042/BSR20192457)
Supplement: Supplementary Figures S1-S2 [file BSR-2019-2457_supp.pdf]

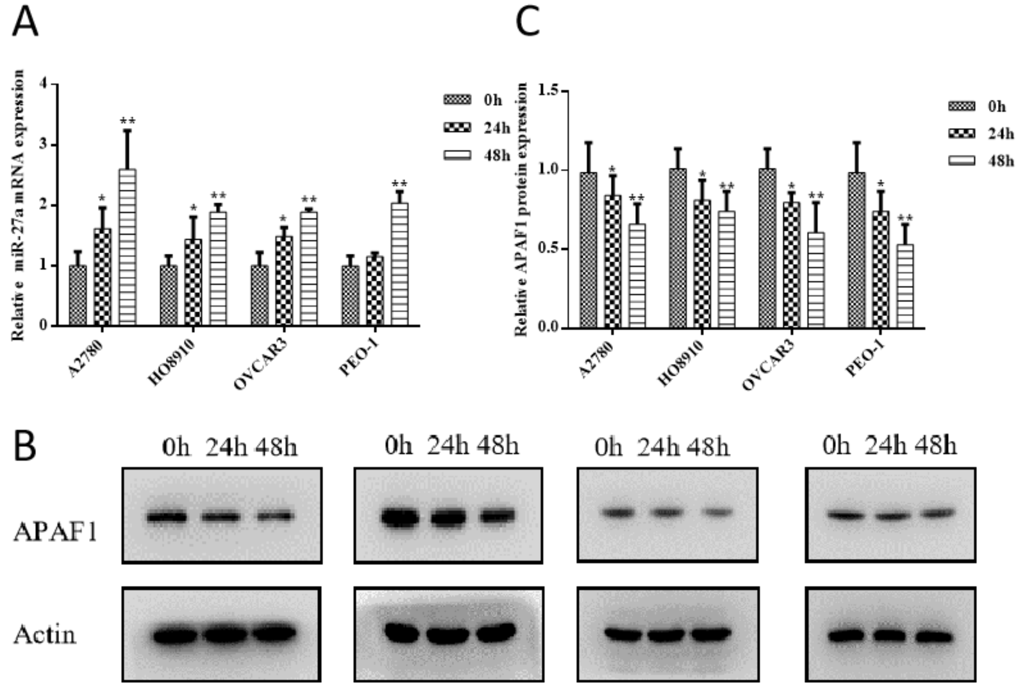

Figure S1. Hypoxia affects the expression of miR-27a and AFAF1 in different OC cell lines. (A) The expression level of miR-27a was detected by qPCR in A2780, HO8910, OVCAR3 and PEO-1 cells when hypoxia treatment for 0h, 24h, 48h. (B) WB analysis the expressionof APAF1 protein inA2780, HO8910, OVCAR3 and PEO-1 cells when hypoxia treatment for 0h, 24h, 48h. (C) Quantify the protein bands of the APAF1 protein (O.D. ratio over β-actin).Data are shown as mean ± SEM (n=3). Asterisks indicate significant differences from the control (*, $p < 0.05$; **, $p < 0.01$; ***, $p < 0.001$, Student's t-test).

Figure S2: APAF1 Full-length sequence >NM_001160.3 Homo sapiens apoptotic peptidase activating factor 1 (APAF1), transcript variant 2, mRNA
AGAGGCGGAGAAGAAGAGGTAGCGAGTGGACGTGACTGCTCTATCCCGGGCAAA
AGGGATAGAACCAGAGGTGGGGAGTCTGGGCAGTCGGCGACCCGCGAAGACTTG
AGGTGCCGCAGCGGCATCCGGAGTAGCGCCGGGCTCCCTCCGGGGTGCAGCCGC
CGTCGGGGGAAGGGCGCCACAGGCCGGGAAGACCTCCTCCCTTTGTGTCCAGTA
GTGGGGTCCACCGGAGGGCGGCCCGTGGGCCGGGCCTCACCGCGGCGCTCCGGG
ACTGTGGGGTCAGGCTGCGTTGGGTGGACGCCCACCTCGCCAACCTTCGGAGGTC
CCTGGGGGTCTTCGTGCGCCCCGGGGCTGCAGAGATCCAGGGGAGGCGCCTGTG
AGGCCCGGACCTGCCCCGGGGCGAAGGGTATGTGGCGAGACAGAGCCCTGCACC
CCTAATTCCCGGTGGAAAACTCCTGTTGCCGTTTCCCTCCACCGGCCTGGAGTCTC
CCAGTCTTGTCCCGGCAGTGCCGCCCTCCCCACTAAGACCTAGGCGCAAAGGCTT
GGCTCATGGTTGACAGCTCAGAGAGAGAAAGATCTGAGGGAAGATGGATGCAAA
AGCTCGAAATTGTTTGCTTCAACATAGAGAAGCTCTGGAAAAGGACATCAAGACA
TCCTACATCATGGATCACATGATTAGTGATGGATTTTTAACAATATCAGAAGAGGAA
AAAGTAAGAAATGAGCCCACTCAACAGCAAAGAGCAGCTATGCTGATTAAAATGA
TACTTAAAAAAGATAATGATTCCTACGTATCATTCTACAATGCTCTACTACATGAAG
GATATAAAGATCTTGCTGCCCTTCTCCATGATGGCATTCCTGTTGTCTCTTCTTCCA
GTGTAAGGACAGTCCTGTGTGAAGGTGGAGTACCACAGAGGCCAGTTGTTTTTGT
CACAAGGAAGAAGCTGGTGAATGCAATTCAGCAGAAGCTCTCCAAATTGAAAGG
TGAACCAGGATGGGTCACCATACATGGAATGGCAGGCTGTGGGAAGTCTGTATTA
GCTGCAGAAGCTGTTAGAGATCATTCCCTTTTAGAAGGTTGTTTCCCAGGGGGAG
TGCATTGGGTTTCAGTTGGGAAACAAGACAAATCTGGGCTTCTGATGAAACTGCA
GAATCTTTGCACACGGTTGGATCAGGATGAGAGTTTTTCCCAGAGGCTTCCACTTA
ATATTGAAGAGGCTAAAGACCGTCTCCGCATTCTGATGCTTCGCAAACACCCAAG
GTCTCTCTTGATCTTGGATGATGTTTGGGACTCTTGGGTGTTGAAAGCTTTTGACA
GTCAGTGTCAGATTCTTCTTACAACCAGAGACAAGAGTGTTACAGATTCAGTAATG
GGTCCTAAATATGTAGTCCCTGTGGAGAGTTCCTTAGGAAAGGAAAAAGGACTTG
AAATTTTATCCCTTTTTGTTAATATGAAGAAGGCAGATTTGCCAGAACAAGCTCATA
GTATTATAAAAGAATGTAAAGGCTCTCCCCTTGTAGTATCTTTAATTGGTGCACTTT
TACGTGATTTTCCCAATCGCTGGGAGTACTACCTCAAACAGCTTCAGAATAAGCAG
TTTAAGAGAATAAGGAAATCTTCGTCTTATGATTATGAGGCTCTAGATGAAGCCATG
TCTATAAGTGTTGAAATGCTCAGAGAAGACATCAAAGATTATTACACAGATCTTTC
CATCCTTCAGAAGGACGTTAAGGTGCCTACAAAGGTGTTATGTATTCTCTGGGACA
TGGAAACTGAAGAAGTTGAAGACATACTGCAGGAGTTTGTAAATAAGTCTCTTTTA
TTCTGTGATCGGAATGGAAAGTCGTTTCGTTATTATTTACATGATCTTCAAGTAGAT
TTTCTTACAGAGAAGAATTGCAGCCAGCTTCAGGATCTACATAAGAAGATAATCAC
TCAGTTTCAGAGATATCACCAGCCGCATACTCTTTCACCAGATCAGGAAGACTGTA
TGTATTGGTACAACTTTCTGGCCTATCACATGGCCAGTGCCAAGATGCACAAGGAA
CTTTGTGCTTTAATGTTTTCCCTGGATTGGATTAAAGCAAAAACAGAACTTGTAGG
CCCTGCTCATCTGATTCATGAATTTGTGGAATACAGACATATACTAGATGAAAAGGA
TTGTGCAGTCAGTGAGAATTTTCAGGAGTTTTTATCTTTAAATGGACACCTTCTTG
GACGACAGCCATTTCCTAATATTGTACAACTGGGTCTCTGTGAGCCGGAAACTTCA

GAAGTTTATCAGCAAGCTAAGCTGCAGGCCAAGCAGGAGGTCGATAATGGAATGC
TTTACCTGGAATGGATAAACAAAAAAAACATCACGAATCTTTCCCGCTTAGTTGTC
CGCCCCCACACAGATGCTGTTTACCATGCCTGCTTTTCTGAGGATGGTCAGAGAAT
AGCTTCTTGTGGAGCTGATAAAACCTTACAGGTGTTCAAAGCTGAAACAGGAGAG
AAACTTCTAGAAATCAAGGCTCATGAGGATGAAGTGCTTTGTTGTGCATTCTCTAC
AGATGACAGATTTATAGCAACCTGCTCAGTGGATAAAAAAGTGAAGATTTGGAATT
CTATGACTGGGGAACTAGTACACACCTATGATGAGCACTCAGAGCAAGTCAATTG
CTGCCATTTCACCAACAGTAGTCATCATCTTCTCTTAGCCACTGGGTCAAGTGACT
GCTTCCTCAAACTTTGGGATTTGAATCAAAAAGAATGTCGAAATACCATGTTTGGT
CATACAAATTCAGTCAATCACTGCAGATTTTCACCAGATGATAAGCTTTTGGCTAGT
TGTTCAGCTGATGGAACCTTAAAGCTTTGGGATGCGACATCAGCAAATGAGAGGA
AAAGCATTAATGTGAAACAGTTCTTCCTAAATTTGGAGGACCCTCAAGAGGATATG
GAAGTGATAGTGAAGTGTTGTTCGTGGTCTGCTGATGGTGCAAGGATAATGGTGG
CAGCAAAAAATAAAATCTTTTTGTGGAATACAGACTCACGTTCAAAGGTGGCTGA
TTGCAGAGGACATTTAAGTTGGGTTCATGGTGTGATGTTTTCTCCTGATGGATCATC
ATTTTTGACATCTTCTGATGACCAGACAATCAGGCTCTGGGAGACAAAGAAAGTAT
GTAAGAACTCTGCTGTAATGTTAAAGCAAGAAGTAGATGTTGTGTTTCAAGAAAA
TGAAGTGATGGTCCTTGCAGTTGACCATATAAGACGTCTGCAACTCATTAATGGAA
GAACAGGTCAGATTGATTATCTGACTGAAGCTCAAGTTAGCTGCTGTTGCTTAAGT
CCACATCTTCAGTACATTGCATTTGGAGATGAAAATGGAGCCATTGAGATTTTAGA
ACTTGTAAACAATAGAATCTTCCAGTCCAGGTTTCAGCACAAGAAAACTGTATGG
CACATCCAGTTCACAGCCGATGAGAAGACTCTTATTTCAAGTTCTGATGATGCTGA
AATTCAGGTATGGAATTGGCAATTGGACAAATGTATCTTTCTACGAGGCCATCAGG
AAACAGTGAAAGACTTTAGACTCTTGAAAAATTCAAGACTGCTTTCTTGGTCATTT
GATGGAACAGTGAAGGTATGGAATATTATTACTGGAAATAAAGAAAAAGACTTTGT
CTGTCACCAGGGTACAGTACTTTCTTGTGACATTTCTCACGATGCTACCAAGTTTT
CATCTACCTCTGCTGACAAGACTGCAAAGATCTGGAGTTTTGATCTCCTTTTGCCA
CTTCATGAATTGAGGGGCCACAACGGCTGTGTGCGCTGCTCTGCCTTCTCTGTGG
ACAGTACCCTGCTGGCAACGGGAGATGACAATGGAGAAATCAGGATATGGAATGT
CTCAAACGGTGAGCTTCTTCATTTGTGTGCTCCGCTTTCAGAAGAAGGAGCTGCT
ACCCATGGAGGCTGGGTGACTGACCTTTGCTTTTCTCCAGATGGCAAAATGCTTAT
CTCTGCTGGAGGATATATTAAGTGGTGGAACGTTGTCACTGGGGAATCCTCACAGA
CCTTCTACACAAATGGAACCAATCTTAAGAAAATACACGTGTCCCCTGACTTCAAA
ACATATGTGACTGTGGATAATCTTGGTATTTTATATATTTTACAGACTTTAGAATAAA
ATAGTTAAGCATTAATGTAGTTGAACTTTTTAAATTTTTGAATTGGAAAAAAATTCT
AATGAAACCCTGATATCAACTTTTTATAAAGCTCTTAATTGTTGTGCAGTATTGCAT
TCATTACAAAAGTGTTTGTGGTTGGATGAATAATATTAATGTAGCTTTTTCCCAAAT
GAACATACCTTTAATCTTGTTTTTCATGATCATCATTAACAGTTTGTCCTTAGGATGC
AAATGAAAATGTGAATACATACCTTGTTGTACTGTTGGTAAAATTCTGTCTTGATGC
ATTCAAAATGGTTGACATAATTAATGAGAAGAATTTGGAAGAAATTGGTATTTTAAT
ACTGTCTGTATTTATTACTGTTATGCAGGCTGTGCCTCAGGGTAGCAGTGGCCTGCT
TTTTGAACCACACTTACCCCAAGGGGGTTTTGTTCTCCTAAATACAATCTTAGAGG
TTTTTTGCACTCTTTAAATTTGCTTTAAAAATATTGTGTCTGTGTGCATAGTCTGCA
GCATTTCCTTTAATTGACTCAATAAGTGAGTCTTGGATTTAGCAGGCCCCCCCACCT

TTTTTTTTTGTTTTTGGAGACAGAGTCTTGCTTTGTTGCCAGGCTGGAGTGCAGTG
GCGCGATCTCGGCTCACCACAATCGCTGCCTCCTGGGTTCAAGCAATTCTCCTGCC
TCAGCCTCCCGAGTAGCTGGGACTACAGGTGTGCGCACATGCCAGGCTAATTTTTG
TATTTTTAGTAGAGACGGGGTTTCACCATGTTGGCCGGGATGGTCTCGATCTCTTG
ACCTCATGATCTACCCGCCTTGGCCTCCCAAAGTGCTGAGATTACAGGCGTGAGCC
ACCGTGCCTGGCCAGGCCCCTTCTCTTTTAATGGAGACAGGGTCTTGCACTATCAC
CCAGGCTGGAGTGCAGTGGCATAATCATACCTCATTGCAGCCTCAGACTCCTGGGT
TCAAGCAATCCTCCTGCCTCAGCCTCCCAAGTAGCTGAGACTACAGGCACGAGCC
ACCACACCCAGCTAATTTTTAAGTTTTCTTGTAGAGACAGGGTCTCACTATGTTGT
CTAGGCTGGTCTTGAACTCTTGGCCTCAAGTAATCCTCCTGCCTCAGCCTCCCAAA
GTGTTGGGATTGCAGATATGAGCCACTGGCCTGGCCTTCAGCAGTTCTTTTTGTGA
AGTAAAACTTGTATGTTGGAAAGAGTAGATTTTATTGGTCTACCCTTTTCTCACTGT
AGCTGCTGGCAGCCCTGTGCCATATCTGGACTCTAGTTGTCAGTATCTGAGTTGGA
CACTATTCCTGCTCCCTCTTGTTTCTTACATATCAGACTTCTTACTTGAATGAAACC
TGATCTTTCCTAATCCTCACTTTTTTCTTTTTTAAAAAGCAGTTTCTCCACTGCTAA
ATGTTAGTCATTGAGGTGGGGCCAATTTTAATCATAAGCCTTAATAAGATTTTTCTA
AGAAATGTGAAATAGAACAATTTTCATCTAATTCCATTTACTTTTAGATGAATGGCA
TTGTGAATGCCATTCTTTTAATGAATTTCAAGAGAATTCTCTGGTTTTCTGTGTAAT
TCCAGATGAGTCACTGTAACTCTAGAAGATTAACCTTCCAGCCAACCTATTTTCCT
TTCCCTTGTCTCTCTCATCCTCTTTTCCTTCCTTCTTTCCTTTCTCTTCTTTTATCTCC
AAGGTTAATCAGGAAAAATAGCTTTTGACAGGGGAAAAAACTCAATAACTAGCTA
TTTTTGACCTCCTGATCAGGAACTTTAGTTGAAGCGTAAATCTAAAGAAACATTTT
CTCTGAAATATATTATTAAGGGCAATGGAGATAAATTAATAGTAGATGTGGTTCCCA
GAAAATATAATCAAAATTCAAAGATTTTTTTTGTTTCTGTAACTGGAACTAAATCAA
ATGATTACTAGTGTTAATAGTAGATAACTTGTTTTTATTGTTGGTGCATATTAGTATA
ACTGTGGGGTAGGTCGGGGAGAGGGTAAGGGAATAGATCACTCAGATGTATTTTA
GATAAGCTATTTAGCCTTTGATGGAATCATAAATACAGTGAATACAATCCTTTGCAT
TGTTAAGGAGGTTTTTTGTTTTTAAATGGTGGGTCAAGGAGCTAGTTTACAGGCTT
ACTGTGATTTAAGCAAATGTGAAAAGTGAAACCTTAATTTTATCAAAAGAAATTTC
TGTAAATGGTATGTCTCCTTAGAATACCCAAATCATAATTTTATTTGTACACACTGTT
AGGGGCTCATCTCATGTAGGCAGAGTATAAAGTATTACCTTTTGGAATTAAAAGCC
ACTGACTGTTATAAAGTATAACAACACACATCAGGTTTTAAAAAGCCTTGAATGGC
CCTTGTCTTAAAAAGAAATTAGGAGCCAGGTGCGGTGGCACGTGCCTGTAATCCC
AGCTCCTTGGGAGGCTAAGACAGGAGGATTCCTTGAGCCCTGGAGTTTGAGTCCA
GCCTGGGTGACATAGCAAGACCCTGTCTTAAAAGAAAAATGGGAAGAAAGACAA
GGTAACATGAAGAAAGAAGAGATACCTAGTATGATGGAGCTGCAAATTTCATGGC
AGTTCATGCAGTCGGTCAAGAGGAGGATTTTGTTTTGTAGTTTGCAGATGAGCATT
TCTAAAGCATTTTCCCTTGCTGTATTTTTTTGTATTATAAATTACATTGGACTTCATAT
ATATAATTTTTTTTTACATTATATGTCTCTTGTATGTTTTGAAACTCTTGTATTTATGAT
ATAGCTTATATGATTTTTTTGCCTTGGTATACATTTTAAAATATGAATTTAAAAAATTT
TTGTAAAAATAAAATTCACAAAATTGTTTTGAAAAACA
